# Preliminary investigation of obstructive sleep apnea and Alzheimer’s disease in down syndrome

**DOI:** 10.1093/sleepadvances/zpaf044

**Published:** 2025-07-23

**Authors:** Dasoo M Yoon, David T Plante, Victoria Fleming, Benjamin Handen, Patrick Lao, Jamie Peven, Bradley Christian, Ozioma Okonkwo, Charles Laymon, Beau Ances, Christy Hom, Brian Helsel, Sigan L Hartley

**Affiliations:** Waisman Center, University of Wisconsin, Madison, Madison, WI 53705, United States; Department of Human Development and Family Studies, University of Wisconsin, Madison 53705, United States; Department of Psychiatry, University of Wisconsin-Madison, Madison, WI 53705, United States; Waisman Center, University of Wisconsin, Madison, Madison, WI 53705, United States; Department of Human Development and Family Studies, University of Wisconsin, Madison 53705, United States; Department of Psychiatry, University of Pittsburgh, Pittsburgh, PA 15213, United States; Taub Institute for Research on Alzheimer’s Disease and the Aging Brain, Sergievsky Center, and Department of Neurology, Vagelos College of Physicians and Surgeons, Columbia University, NY, New York 10032, United States; Department of Psychiatry, University of Pittsburgh, Pittsburgh, PA 15213, United States; VA Pittsburgh Healthcare System, Pittsburgh, PA 15240, United States; Waisman Center, University of Wisconsin, Madison, Madison, WI 53705, United States; Department of Psychiatry, University of Wisconsin-Madison, Madison, WI 53705, United States; School of Medicine, University of Wisconsin-Madison, Madison, WI 53705, United States; Department of Radiology, University of Pittsburgh, Pittsburgh, PA 15213, United States; Department of Bioengineering, University of Pittsburgh, Pittsburgh, PA 15260, United States; Department of Neurology, Washington University School of Medicine in St. Louis, St. Louis, MO 63130, United States; Department of Psychiatry & Human Behavior, University of California, Irvine, Orange, CA 92868, United States; Department of Neurology, University of Kansas Medical Center, Kansas City, KS 66160, United States; Waisman Center, University of Wisconsin, Madison, Madison, WI 53705, United States; Department of Human Development and Family Studies, University of Wisconsin, Madison 53705, United States

**Keywords:** down syndrome, obstructive sleep apnea, Alzheimer’s disease, sleep, biomarkers, dementia, cognitive impairment, depression, amyloid

## Abstract

This study provided a preliminary examination of indices of obstructive sleep apsnea (OSA) and sleep disruptions in adults with Down syndrome (DS), and their associations with Alzheimer’s disease (AD) pathology and symptomatology. A total of 93 adults with DS (aged 25–61 years) from the Alzheimer Biomarker Consortium—DS completed cognitive assessments, MRI and positron emission tomography (PET) scans (assessing amyloid-beta [Aβ] and tau), and a one-night home sleep study using the WatchPAT-300 device. Study partners also reported on depressive symptoms and diagnoses. Correlational analyses examined relationships between sleep variables, PET biomarkers, and AD symptomatology (cognitive functioning and depressive mood), controlling for sociodemographics. A total of 81 participants (87 per cent) completed valid WatchPAT data. Of these, 60 (74 per cent) screened positive for OSA, and an additional 11 had a prior OSA diagnosis and used CPAP during the test night. Nearly half (45 per cent) of those screening positive for OSA had no prior diagnosis, indicating under-detection. Among the 22 participants using OSA treatment, 50 per cent continued to show sleep-disordered breathing, suggesting suboptimal treatment effectiveness. Higher wake percentage and shorter total sleep time were associated with greater Aβ and tau burden. Cognitive performance was negatively associated with wake percentage, total sleep time, and oxygenation indices (minimum oxygen, desaturation, and time ≤ 88 per cent oxygen). Depressive symptoms were negatively related to total sleep time. These findings add preliminary evidence linking sleep disruption and OSA with AD-related pathology and symptomatology. Larger, longitudinal studies are needed to confirm these associations and evaluate whether improving sleep quality and treating OSA may help delay AD onset in this high-risk population.

**Statement of Significance**

These preliminary findings suggest that sleep problems may often go undetected and that treatment compliance may be suboptimal in adults with DS, highlighting the potential need for developing screening approaches and management strategies in this population. However, given the modest sample size and cross-sectional design, these results should be interpreted with caution, and further research is needed to inform evidence-based guidelines. Our findings add to the preliminary evidence linking OSA and sleep disturbances with AD pathology and symptomatology in DS, suggesting that OSA may represent a modifiable target for interventions aimed at delaying AD onset in this high-risk population.

## Introduction

Down syndrome (DS) is a genetic condition caused by the triplication of chromosome 21, affecting 1 in 1000 births worldwide [1, 2]. Individuals with DS experience a range of sleep problems across their lifespan, one of the most prevalent being obstructive sleep apnea (OSA), a condition characterized by repeated episodes of apneas (complete upper airway obstructions) and hypopneas (partial obstructions) [3–5]. Anatomical and physiological characteristics unique to DS including midface hypoplasia, enlarged tonsils and adenoids, macroglossia, and co-occurring medical conditions such as obesity and hypothyroidism are thought to contribute to OSA in DS [5–8]. The rate of OSA is reported to be 30 per cent to 50 per cent in children with DS and 40 per cent to 94 per cent in adults with DS. However, much of the existing research on OSA in DS is based on informant reports of problems or clinical diagnosis as many individuals with DS may not be regularly evaluated for OSA given the burden of in-lab sleep studies [5, 9–12]. In addition, prior reports suggest that compliance with prescribed continuous positive airway pressure (CPAP) devices or other OSA treatments is difficult in individuals with DS [13]. Thus, even when diagnosed, individuals with DS may often continue to experience significant sleep disruptions and sleep-disordered breathing problems in everyday life.

In the general population and outside of DS, OSA, and sleep disruptions are associated with an increased risk of Alzheimer’s disease (AD), through oxidative stress, metabolic and vascular deregulation, increased inflammation, and by altering amyloid-beta (Aβ) and tau production, phosphorylation, and clearance [11, 14, 15]. Individuals with DS have a 90 per cent lifetime risk of AD with an average age of dementia onset of 55 years, but onset is reported to range from the late 30s to 70+ years [16, 17]. The high risk for AD in DS is posited to be primarily driven by the triplication of the amyloid precursor protein (APP) gene, which leads to the overproduction of Aβ [18]. The accumulation of Aβ plaques, followed by tau neurofibrillary tangles, are early features in the sequence of pathology that precede AD dementia onset in both the general population and in individuals with DS [17–19]. It is not known if OSA or sleep disruptions are related to heterogeneity in the timing of AD pathology in individuals with DS.

Studies using polysomnography and wrist-worn actigraphy have shown that adults with DS exhibit low sleep efficiency, prolonged wake after sleep onset, and frequent nighttime awakenings [10, 20]. These sleep disruptions are more pronounced compared to age-, sex-, and BMI-matched controls without DS. Furthermore, in a prior study by Cody et al., longer nighttime awakenings were reported to be associated with higher positron emission tomography (PET) Aβ burden and lower memory and cognitive performance in a sample of 47 adults with DS [38]. These findings suggest that sleep disruptions may play a role in accelerating the AD pathology in individuals with DS.

Given the high prevalence of OSA and the elevated risk for AD in individuals with DS, there is a critical need to understand whether OSA or sleep disruptions are related to elevated levels of Aβ or tau burden or AD symptomatology in models controlling for age in adults with DS. The present study sought to provide a preliminary examination of OSA and sleep disruptions using an objective home sleep test in a sample of 81 adults with DS (aged 25–61 years) and evaluated their associations with PET Aβ and tau burden and direct and informant-based measures of early AD symptomatology, which have been previously shown to include impairments in memory, executive functioning, and motor planning and control and depressed mood [21–23]. The study aims were: (1) to characterize real-life sleep disruptions and sleep-disordered breathing in a cohort of adults with DS to understand the prevalence, under-detection, and under-treatment of OSA and its association with sociodemographic variables; and (2) examine the relation between sleep disruptions and sleep-disordered breathing and AD pathology (PET Aβ and tau) and symptomatology (cognitive performance and depressed mood). Based on prior research [9, 10, 19], we hypothesized that there would be a high overall prevalence of OSA in the cohort of adults with DS and, given challenges in diagnosing OSA in this population, a high number of adults with DS without a prior OSA diagnosis would screen positive for OSA using the WatchPAT device. In line with reports of low compliance with CPAP treatments [13]. Finally, we hypothesized that greater sleep disruptions and higher OSA severity would be associated with higher PET Aβ and tau burden, lower cognitive performance, more depressed mood symptoms, and the presence of depressive disorder.

## Methods

### Participants

A total of 93 adults with DS who were enrolled in the Alzheimer Biomarker Consortium—Down Syndrome (ABC-DS) at three sites—University of Wisconsin-Madison, University of Pittsburgh, and Washington University-St. Louis—consented to participate in an auxiliary study examining lifestyle factors. Inclusion criteria for ABC-DS were as follows: (1) age ≥25 years, (2) karyotype indicative of DS (full trisomy, mosaicism, or translocation), (3) mental age ≥3 years, (4) no contraindications for brain MRI or PET imaging (e.g. pregnancy, metallic implants), and (5) no untreated medical or psychiatric conditions affecting cognition. For the auxiliary lifestyle study, participants could not have a diagnosis of AD dementia at the time of study entry. Each participant was accompanied by a study partner—a family member or staff member with regular contact with the adult with DS—who provided additional informant data. Participants who used CPAP or other OSA treatments were included to capture real-world sleep patterns, given that many adults with DS struggle with CPAP compliance and/or may have suboptimal settings on CPAP devices as in-lab sleep studies can be burdensome and may be avoided in this population [10, 12, 24].

## Procedure

As part of the core ABC-DS protocol, participants underwent a 2.5-h battery of cognitive measures and brain imaging scans (MRI and PET) to assess AD biomarkers (see Handen et al. [25], p.6, for a full list of activities). Study partners completed informant measures about the participant’s sociodemographics, medical history, dementia symptoms, and mood. For the auxiliary study, participants wore the WatchPAT-300 (WatchPAT; Itamar Medical Inc., Atlanta, GA) device for one night to objectively measure sleep-disordered breathing and sleep patterns. Study partners and participants with DS also completed a sleep log, including information including time in and out of bed, to help validate and interpret WatchPAT data.

## Measures

### Sociodemographics and control variables

Study partners reported the age of the adult with DS (coded in years) and their biological sex (coded: 1 = male, 2 = female). Study partners also reported the presence of a medical diagnosis of OSA (coded: 0 = no OSA diagnosis, 1 = diagnosis of OSA) and whether the participant was receiving treatment for OSA (coded: 1 = yes, 2 = no, 3 = unknown). BMI was calculated as weight in kilograms (kg) divided by height in meters squared (m^2^). The level of premorbid intellectual disability (ID) was determined based on standardized IQ (Kaufman Brief Intelligence Test [KBIT-2] [26] or Stanford-Binet, Fifth Edition [27], and adaptive behavior assessment conducted prior to any concerns of mild cognitive impairment (MCI) or dementia. Level of ID was coded as mild, moderate, or severe/profound based on IQ standard scores (mild: 50–70, moderate: 35–49, and severe/profound: <35) or age equivalent scores (mild: 9–14 years, moderate: 4–8 years, and severe/profound: ≤3 years).

Clinical AD status was determined through a case consensus process involving a multidisciplinary team of at least two research staff experienced in AD and DS, a licensed psychologist, and a physician. The consensus process involved a review of directly administered measures of cognitive function; study partner-reported measures of behavior, functioning, and dementia symptoms; a neurological exam; and medical/psychiatric history. The team conducting the consensus review was blind to neuroimaging, genetic, and biofluid biomarker data. Participants were classified into one of four categories: (1) Cognitively Stable: The participant remained at their baseline cognitive functioning; (2) MCI: The participant exhibited mild cognitive decline with limited impact on daily functioning; (3) Dementia: The participant demonstrated significant cognitive decline and impairment in daily functioning; (4) Unable to Determine: Clinical status could not be determined due to testing challenges, medical or psychiatric conditions, or major life events.

### Cognitive functioning

Performance on three directly administered cognitive measures was analyzed. These measures were selected because they assess cognitive domains (memory, executive functioning, and motor control and planning) known to decline early in the progression of AD in DS [28–30]. The Modified Cued Recall Test (mCRT) evaluates learning and episodic verbal memory by presenting participants with 12 pictures of items across three learning trials, each accompanied by a category cue (e.g. “fruit” for a picture of grapes) [31]. During the subsequent testing phase, participants are first asked to recall as many pictures as possible (free recall), followed by category cues for any missed items (cued recall). A total of three test trials are conducted. The total mCRT score is the sum of items recalled during both free and cued trials, ranging from 0 to 36. The mCRT has demonstrated good inter-test reliability among adults with DS, with satisfactory reliability coefficients for both free recall (α = 0.814) and total scores (α = 0.709) [31]. Moreover, the mCRT has been associated with biomarkers of early AD pathology, including elevated PET Aβ and tau levels [32, 33].

The Cat and Dog Modified Stroop Task (CatDog) is an adaption of the day-night Stroop task and assesses executive functioning, including response inhibition, working memory, and rule maintenance [34, 35]. During the Naming Trial, participants view a series of 16 pictures of cats and dogs and are tasked with correctly naming them (i.e. “cat” or “dog”) as quickly as possible. In the Switch Trial, participants repeat the task but must switch the names of the pictures (e.g. say “cat” when referring to a picture of a dog). Both the time taken in seconds and the number of errors are recorded for each trial. The validity of the task as a measure of executive functioning and inhibition has been supported by previous studies, with further established reliability, especially in DS populations [34–36].

The Purdue Pegboard (Pegboard) test evaluates fine and gross manual dexterity, as well as motor coordination [37]. It has been established as one of the popular dexterity tests for individuals with DS [38]. Participants are required to place as many pegs as possible in the corresponding column within a 30-s timeframe. The dominant hand score was used. The test has moderate to high test–retest reliability among adolescents and young adults with DS, with an intra-class correlation coefficient (ICC) of 0.924 (95 per cent CI: 0.773 to 0.974, *p*<.001) for the dominant hand [38].

### Depressed mood symptoms

Depressed mood symptoms were also assessed as they have also been shown to present early on in AD symptomatology [39]. These symptoms were assessed through the Reiss Screen for Maladaptive Behavior (Reiss), a 38-item informant report of maladaptive behaviors in people with intellectual disability [40]. Analyses focused on two of the eight subscales: Depression – Behavioral Signs (Depression-B) and Depression—Physical Signs (Depression-P). Items are rated on a three-point scale as “no problem,” (coded 0) “problem,” (coded 1), or “major problem” (coded 2) and summed for each domain. The Reiss has demonstrated good inter-rater reliability (*r* = 0.67), test–retest reliability (*r* = 0.75), and internal consistency (α = 0.85) [41]. Study partners also reported on the presence (versus absence) of a depression diagnosis by a health provider (coded: 1 = diagnosed with depressive disorder; 0 = not diagnosed).

### Sleep-disordered breathing

The WatchPAT 300, a clinically validated home sleep test that measures arterial pulsatile volume changes, heart rate, oximetry, actigraphy, and chest motion, was used over one night to assess disrupted sleep and sleep-disordered breathing. The device includes a finger probe that collects Peripheral Arterial Tonometry (PAT) signals and oxygen saturation levels, a sensor positioned under the sternal notch that collects snoring and body position data, and an accelerometer embedded in the wristband unit. Raw data were manually reviewed and edited by a board-certified sleep physician who made final determinations on OSA levels. Apneas and hypoapneas were quantified requiring a 4 per cent or greater oxygen desaturation from baseline. The WatchPAT 300 device has been shown to be valid in populations at high risk for OSA including clinical populations and populations with elevated BMI and other metabolic conditions [42, 43]. Key variables analyzed included: Total Sleep Time (TST), Percent of Wake State (%Wake), Percent of Sleep with O_2_ Saturation ≤88 per cent (% ≤88), Minimum Blood Oxygen Levels (MinOxy), Mean Desaturation Nadir (MDesat), and PAT Apnea-Hypopnea Index (pAHI).

### Biomarkers of AD pathology

MRI scans were conducted on 3.0 Tesla scanners across all three sites – GE Discovery MR750 (Wisconsin), Siemens Prisma (Pittsburgh), and Siemens Prisma (Washington-St. Louis). Consistent with the AD Neuroimaging Initiative and Human Connectome Project protocols, high-resolution T1-weighted images were acquired using either a 3-dimensional fast spoiled gradient echo sequence or magnetization-prepared rapid-acquisition gradient echo sequence. Freesurfer 5.3 and Desikan-Killany atlases were used to process the PET images [44]. Two PET tracers were used to measure AD pathology: [^11^C]PiB for Aβ burden and [^18^F]AV-1451 for tau burden.

Images were obtained post-injection at 50–70 min for [^11^C]PiB and 80–100 min for [^18^F]AV-1451. An iterative method was used for data reconstruction, with corrections applied for deadtime, attenuation, scatter, and radioactive decay. A frame-by-frame approach was applied to correct for motion in the 5-min frames. For Aβ burden, units of centiloids were calculated from the [^11^C]PiB data using standardized published methods [45]. For tau burden, SUVR (standardized uptake value-ratio) outcomes were determined using volume-weighted average of [^18^F]AV-1451 radiotracer concentration within the regional parcellations of the T1W MRI using the composite region defined by Jack et al. divided by the cerebellar cortex concentration [46].

**Figure 1:**
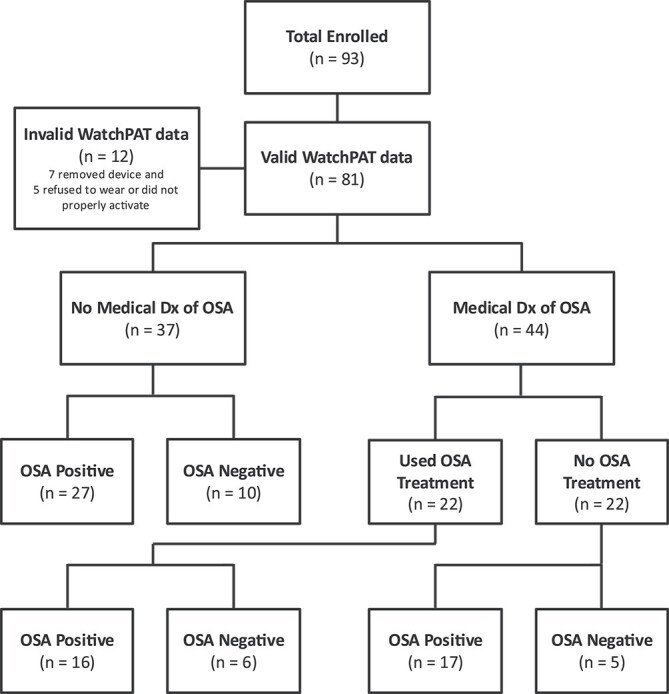
Participant flow and classification by OSA diagnosis and treatment status. Flow of participants through the study and breakdown of OSA screening results based on the WatchPAT home sleep study across groups defined by prior OSA diagnosis and treatment use.

### Data analysis

Histograms and descriptive statistics were used to examine score distributions and identify outliers for WatchPAT, cognitive function, and PET biomarker outcomes. Pearson correlations and independent sample t-tests examined the relation between sociodemographics and WatchPAT variables, to understand any differences in OSA by sociodemographic factors. Sociodemographics and scores on measures of AD pathology (PET Aβ and tau) and symptomatology (cognitive performance and depressive symptoms or depressive disorder), were then compared between the group of participants who screened positive for OSA versus those who screened negative on the WatchPAT home sleep study. Partial correlations, controlling for age, sex, and premorbid ID level, examined the association between WatchPAT variables and biomarkers of AD pathology and AD symptomatology. For the majority of participants (n = 66, 77 per cent) all study variables were collected at the same study visit. However, there was up to a 6-month lag between sleep, mood, and cognitive data collection and MRI and PET scans for 18 (23 per cent) participants. A time lag variable was originally included in models but did not impact finding and thus was removed. Finally, follow-up analyses were conducted using only the subset of participants who did not use OSA treatment on the night of the WatchPAT home sleep study to ensure that OSA treatment was not obscuring an actual relationship between sleep-disordered breathing and OSA and AD pathology and symptomatology. For all analyses, an alpha level of .05 was used to determine statistical significance.

## Results

As shown in Figure 1, of the 93 participants, valid WatchPAT data were obtained from 81 (87 per cent) participants. Seven (8 per cent) participants removed the device prematurely before sufficient data was collected and five (5 per cent) participants either failed to activate the device correctly or refused to wear it. Table 1 displays the sociodemographics of the 81 participants with valid WatchPAT data. On average, participants were 38.3 years old (SD = 8.0), with approximately half identifying as female (n = 37, 46 per cent). The majority of participants had mild (n = 31, 38 per cent) or moderate (n = 35, 43 per cent) levels of ID. Three (4 per cent) participants had a clinical status of MCI while the rest were cognitively stable. Of the 81 participants with valid WatchPAT home sleep study data, 44 (54.3 per cent) had a prior medical diagnosis of OSA and 37 (45.7 per cent) did not.

**Table 1:** Participant sociodemographics and means and standard deviations for primary study variables

	Variable	Total	WatchPAT Positive for OSA	WatchPAT Negative for OSA	t or χ^2^ value
		N = 81	N = 60	n = 21	
Socio-demographics	Age in Years, M (SD)	38.26 (8.01)	38.33 (7.59)	38.05 (9.30)	−0.14
	Sex, N (%)				0.513
	Male	44 (54.32)	34 (56.7)	10 (47.6)	
	Female	37 (46.68)	26 (43.4)	11 (52.4)	
	Race, N (%)				3.18
	White	78 (97.5)	58 (96.7)	20 (95.2)	
	Black	1 (1.3)	0 (0)	1 (4.8)	
	Unknown/Unreported	1 (1.3)	1 (1.7)	0 (0)	
	BMI, M (SD)	33.19 (7.55)	33.78 (7.18)	30.84 (8.91)	−1.102
	Premorbid ID, N (%)				1.253
	Mild	31 (38.27)	25 (41.7)	6 (28.6)	
	Moderate	35 (43.21)	24 (40.0)	11 (52.4)	
	Severe	15 (18.52)	11 (18.3)	4 (19.0)	
Cognitive Performance	CatDog Naming Time, (SD)	14.37 (7.25)	14.75 (8.16)	12.28 (3.46)	−0.798
	CatDog Naming Errors, M (SD)	0.10 (0.50)	.08 (.424)	.14 (.66)	0.477
	CatDog Switching Time, M (SD)	25.28 (15.49)	26.41 (17.44)	22.08 (7.18)	–1.102^*^
	CatDog Switching Errors, M (SD)	1.59 (3.39)	1.86 (3.74)	.81 (1.94)	–1.230^*^
	mCRT, M (SD)	31.79 (6.26)	31.70 (6.87)	32.05 (4.20)	0.218
	Purdue Pegboard D, M (SD)	7.10 (2.21)	6.97 (2.31)	7.71 (1.70)	0.805
Depressive Mood	Reiss Depression – P, M (SD)	0.74 (1.39)	.83 (1.55)	.48 (.75)	−1.01
	Reiss Depression – B, M (SD)	0.48 (0.91)	.57 (.98)	.24 (.625)	−1.434^*^
	Depression Dx N (%)	7 (9)	7 (11.7)	0 (0.00)	2.557
WatchPAT	Total Sleep (min), M(SD)	478.95 (70.71)	484.58 (73.01)	456.40 (57.33)	−1.389
	% Wake, M(SD)	12.36 (8.69)	12.08 (9.16)	13.46 (6.64)	0.549
	% ≤88, M(SD)	0.56 (4.06)	.70 (4.54)	0 (.00)	−0.592
	Oxygen Minimum, M(SD)	82.27 (8.47)	80.58 (8.60)	88.93 (2.82)	3.697^*^
	Mean Desaturations, M(SD)	90.88 (3.20)	90.45 (3.37)	92.71 (1.20)	2.465
	pAHI, M(SD)	19.34 (19.23)	23.49 (19.37)	2.71 (1.57)	−4.129^*^^*^
	Severity of OSA, N(%)				81^*^^*^
	None	21 (25.93)	0 (0)	21 (100)	
	Mild	24 (29.63)	24 (40.0)	0 (0)	
	Moderate	18 (22.22)	18 (30.0)	0 (0)	
	Severe	18 (22.22)	18 (30.0)	0 (0)	

Note: ^*^ = p < .05, ^*^^*^ = p < 0.001. Includes means (SDs) and frequencies (%) for the total sample and for participants who screened positive vs. negative for OSA on WatchPAT. Also includes t or χ^2^ test values comparing groups.

### Sleep disruptions and OSA indices

Of the 81 participants with valid sleep data, 60 (74 per cent) screened positive for OSA based on the WatchPAT home sleep study. Severity of OSA was determined using pAHI, which represents the number of apneas and hypoapneas per hour (<5 = No OSA, 5–14 = Mild OSA, 15–29 = Moderate OSA, ≥30 = Severe OSA). Of the 60 participants who screened positive for OSA on the WatchPAT, 24 (30 per cent) had mild OSA, 18 (22 per cent) had moderate OSA, and 18 (22 per cent) had severe OSA. Of these 60 participants, 27 (45 per cent) did not have a prior medical diagnosis of OSA. The remaining 33 (55 per cent) participants had a prior medical diagnosis of OSA. The majority (n = 23, 70 per cent) of the 33 participants with a prior medical diagnosis of OSA did not use OSA treatments on typical nights nor during the WatchPAT home sleep study. However, a subset (n = 10, 30 per cent) used a PAP device (n = 9; 27 per cent) or mandibular advancing oral appliance (n = 1, 3 per cent) on typical nights and during the WatchPAT home sleep study. An additional 11 participants had a prior medical diagnosis of OSA but screened negative for OSA on the WatchPAT home sleep study; all 11 of these participants were using a PAP device on the home sleep study, suggesting that their OSA was successfully managed. The chi-square test revealed no significant differences in OSA severity levels (none, mild, moderate, severe) between participants with versus without a prior medical diagnosis of OSA (χ^2^ = 1.398, *p*=.706).

Table 1 also presents differences between participants who screened positive versus negative for OSA according to the WatchPAT. There were no statistically significant differences in sociodemographic characteristics, including age, sex, race, BMI, and premorbid intellectual disability level. Participants who screened positive for OSA had significantly lower minimum oxygen levels during sleep compared to those who screened negative, t(79) = 3.70, *p*=.003. They also exhibited significantly higher pAHI scores than the OSA-negative group, t(79) = –4.13, *p*<.001. In addition, the OSA-positive group showed significantly longer CatDog Switching Time, t(79) = –1.10, *p*=.026, made more Switching Errors, t(79) = –1.23, *p*=.032, and demonstrated higher behavioral signs of depression on the Reiss Depression–B subscale, t(79) = –1.43, *p*=.034.

### OSA and sleep disruptions and biomarkers of AD pathology

Correlations were first conducted to identify sociodemographic variables related to WatchPAT indices to determine if sleep disruptions and OSA differed by sociodemographics. Table 2 shows these correlations for the full sample (n = 81). BMI was significantly negatively correlated with MinOxy (r = –0.314, *p*=.036) and positively correlated with pAHI (r = 0.299, *p*=.043). There were no significant associations between age, sex, or premorbid intellectual disability level and WatchPAT sleep variables. Follow-up analyses were conducted on the subset of 60 participants not using OSA treatments. As shown in Table 3, BMI continued to be significantly negatively associated with MinOxy (r = –0.451, *p*=.004), and positively associated with pAHI (r = 0.481, *p*=.002). Additionally, having APOE-ε4 was significantly associated with higher MinOxy (r = 0.310, *p*=.048).

**Table 2:** Correlations between WatchPAT variables and sociodemographics

	TST	%Wake	% ≤88	MinOxy	MDesat	pAHI
Age	–.094, .421	.037, .754	.010,.931	–.084, .476	–.013, .913	.068, .563
Sex	–.072, .537	.116, .322	–.093, .425	–.021, .860	.121, .303	–.099, .400
Race	.134, .256	–.085, .473	–.016, .890	–.100, .398	–.032, .786	**.269, .020**
BMI	.002, .990	.103, .494	–.080, .595	**–.314, .036**	–.186, .216	.**299, .043**
APOE-ε4	–.047, .779	.162, .332	–.078,.640	.292, .075	.217, .190	–.181, .278
Premorbid ID	–.040, .735	–.024, .838	.027,.818	.061, .603	–.071, .550	–.076, .515

Note: Bold values = p < .05. TST = total sleep time, %Wake = percent of wake state, %≤88 = percent of sleep with O_2_ Saturation ≤88%, MinOxy = minimum oxygen level, pAHI = PAT apnea-hypoapnea index.

Pearson correlation coefficients examining associations between demographic factors and study outcomes.

**Table 3:** Correlations between WatchPAT variables and sociodemographics (Subset with no OSA treatment)

	TST	%Wake	% ≤88	MinOxy	MDesat	pAHI
Age	–.212, .060	**.249, .033**	.013, .462	–.128, .178	–.033, .406	.092, .253
Sex	–.160, .121	.197, .074	–.118, .196	.072, .304	.156, .127	–.203, .069
Race	–	–	–	–	–	**–**
BMI	–.036, .419	.171, .166	–.056, .377	**–.451, .004**	–.253, .075	**.481, .002**
APOE-ε4	.082, .333	.040, .416	–.091, .315	**.310, .048**	.234, .107	–.184, .166
Premorbid ID	–.058, .336	–.113, .206	.066, .315	.053, .353	–.103, .228	.041, .384

Note: Bold values = p < .05. TST = total sleep time, %Wake = percent of wake state, %≤88 = percent of sleep with O_2_ Saturation ≤88%, MinOxy = minimum oxygen level, pAHI = PAT apnea-hypoapnea index.

Same as Table 2, but limited to participants not using OSA treatment.

**Figure 2:**
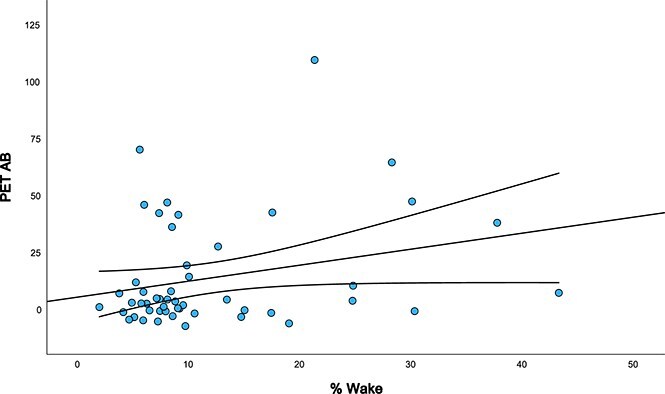
Association between % wake and PET aβ. Scatterplot illustrating the association between percentage of wake time during the night (% wake) and amyloid-beta burden (PET aβ) among adults with DS. Regression line and 95 per cent confidence intervals included.

**Figure 3:**
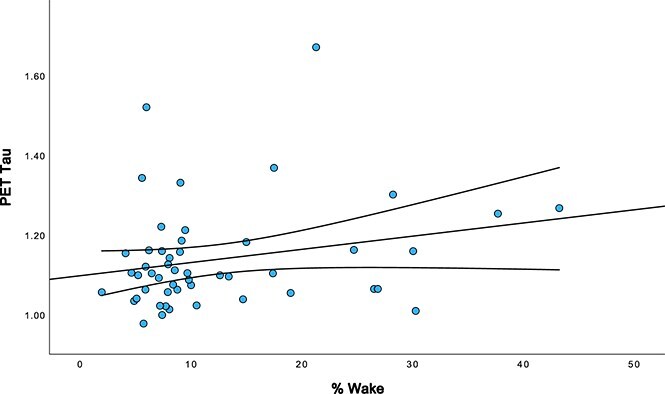
Association between % wake and PET tau. Scatterplot illustrating the association between percentage of wake time during the night (% wake) and tau burden (PET tau) among adults with DS. Regression line and 95 per cent confidence intervals included.

Table 4 presents the partial correlations (controlling for age, sex, and premorbid ID level) between WatchPAT variables and PET Aβ and tau for the full sample of 81. There was a significant positive association between %Wake and PET Aβ (r = 0.302, *p*=.021) and PET tau (r = 0.273, *p*=.033). These associations are illustrated in Figures 2 and 3. TST was negatively correlated with PET Aβ at a trend level (r = –0.241, *p*=.053). Follow-up analyses were conducted to re-run partial correlations on the subset of 60 participants not receiving OSA treatment (Table 5). In these follow-up analyses, %Wake was related to PET Aβ at a trend-level (r = 0.259, *p*=.056) and there remained a significant association between %Wake and PET tau (r = 0.302, *p*=.031). There was also a trend-level positive association between % ≤ 88 and PET tau burden (r = 0.242, *p*=.069).

**Table 4:** Partial Correlations between WatchPAT and AD biomarkers, cognitive impairments, and depression

	TST	% Wake	% ≤88	MinOxy	MDesat	pAHI
PET Aβ	–.241+, .053	**.302, .021**	–.067, .650	.114, .435	.151, .300	–.141, .334
PET Tau	–.124, .205	**.273, .033**	.228, .115	–.241, .096	–.100, .496	.078, .594
CatDog Naming Time	–.183+,.063	**.227, .029**	–.048, .347	.022, .848	–.011, .463	.014, .454
CatDog Naming Error	**–.241, .021**	.181+, .066	.123, .154	–.024, .835	.087, .237	–.100, .203
CatDog Switch Time	.013, .458	.087, .235	**–.269, .012**	.197, .092	**–.197, .050**	.164, .086
CatDog Switch Error	–.005, .482	.050, .341	**–.238, .023**	.093, .428	–.168+, .080	.057, .318
mCRT	.085, .240	–.087, .233	.024, .420	–.110, .180	–.085, .239	.057, .317
Pegboard	**.381, .010**	–.266+,.056	.**306, .033**	**–.325, .047**	**.330, .023**	–.176,.149
Reiss Depression-B	**–.552, <.001**	**.313, .043**	–.130, .244	.201, .140	.108, .281	–.208, .131
Reiss Depression-P	**–.440, .007**	.178, .169	.043, .409	.040, .415	–.093, .310	–.020, .457
Depression Dx	**–.309, .046**	.103, .291	–.089, .317	–.022, .454	–.013, .473	–.065, .364

Note: Associations were adjusted for relevant sociodemographics. Bold = p ≤ .05. + = p < .08. TST = total sleep time, %Wake = percent of wake state, %≤88 = percent of sleep with O_2_ Saturation ≤88%, MinOxymin= minimum oxygen level, pAHI = PAT apnea-hypoapnea index.

Partial correlations controlling for age, sex, and premorbid ID level.

**Table 5:** Partial Correlations between WatchPAT and AD biomarkers, cognitive impairments, and depression (Subset with no OSA Treatment)

	TST	% Wake	% ≤88	MinOxy	MDesat	pAHI
PET Aβ	–.150, .181	.259+, .056	–.072, .333	.088, .296	.144, .191	–.121, .231
PET Tau	–.083, .308	**.302, .031**	.242+, .069	–.227, .083	–.093, .288	.054, .373
CatDog Naming Time	–.242, .103	**.426, .011**	.131, .249	**–.503, .003**	–.273+, .076	**.412, .013**
CatDog Naming Error	–.101, .300	.137, .239	–.036, .426	.125, .258	.083, .334	–.130, .251
CatDog Switch Time	–.117, .272	.308+, .052	**.347, .033**	**–.540, .001**	**–.425, .011**	**.448, .007**
CatDog Switch Error	–.248, .098	.275+, .074	.170, .189	–.217, .129	–.168, .192	.132, .247
mCRT	–.130, .250	.209, .138	.017, .465	.024, .450	–.053, .392	–.038, .422
Pegboard	.297+, .059	–.174, .183	**–.423, .011**	**.460, .006**	**.440, .008**	**–.499, .003**
Reiss Depression-B	–.180, .175	.004, .492	–.110, .284	.196, .154	.096, .311	–.167, .194
Reiss Depression-P	–.248, .97	.069, .361	.120, .268	.091, .319	–.113, .279	–.008, .483
Depression Dx	–.049, .401	–.015, .469	–.067, .365	.005, .490	–.032, .434	–.029, .441

Note: Associations were adjusted for relevant sociodemographics. Bold = p ≤ .05. + = p < .08. TST = total sleep time, %Wake = percent of wake state, %≤88 = percent of sleep with O_2_ Saturation ≤88%, MinOxy = minimum oxygen level, pAHI = PAT apnea-hypoapnea index.

Same structure as Table 4, limited to participants not receiving OSA treatment.

### OSA and sleep disruptions and AD symptomatology

**Figure 4:**
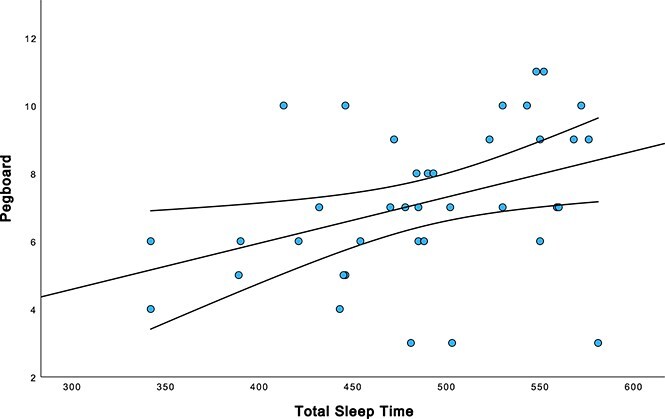
Association between Total sleep time and pegboard performance. Scatterplot depicting the association between total sleep time (minutes) and pegboard performance (dominant hand score). Increased total sleep time is associated with better fine motor performance. Regression line and 95 per cent confidence intervals included.

Table 4 also presents the partial correlations (controlling age, sex, and premorbid ID) between WatchPAT variables and AD symptomatology for the full sample (n = 81). CatDog Naming Time was significantly positively correlated with %Wake (r = 0.227, *p* = .029), while CatDog Naming Errors was significantly negatively correlated with TST (r = –0.241, *p* = .021). CatDog Switch Time was significantly negatively correlated with % ≤88 (r = –0.269, *p* = .012) and MDesat (r = –0.197, *p* = .050), while the CatDog Switch Errors was also significantly negatively correlated with % ≤88 (r = –0.238, *p* = .023) and negatively associated at the trend level with MDesat (r = –0.168, 0.080). Pegboard was significantly positively correlated with TST (r = 0.381, *p* = .010), % ≤88 (r = 0.036, *p* = .033), and MDesat (r = 0.330, 0.023) and significantly negatively correlated with MinOxy (r = –0.325, *p* = .047) and at the trend level with %Wake (r = –0.266, *p* = .056). The association between total sleep time and Pegboard performance is illustrated in Figure 4. There were no other significant correlations between WatchPAT variables and cognitive scores.

In follow-up analyses that included the subset of 60 participants who did not use OSA treatment, the same pattern of findings emerged (Table 5). Specifically, CatDog Naming Time was significantly negatively associated with MinOxy (r = –0.503, *p* = .003) and MDeSat (r = –0.273, *p* = .076) at the trend level, and significantly positively associated with pAHI (r = 0.412, *p* = .013). CatDog Naming Time was significantly positively correlated with %Wake (r = 0.426, *p* = .011) and pAHI (r = 0.412, *p* = .013) and negatively correlated with MinOxy (r = –0.503, *p* = .003). CatDog Switch Time was significantly positively correlated with % ≤88 (r = 0.347, *p* = .033) and pAHI (r = 0.448, *p* = .007) and negatively correlated with MinOxy (r = –0.540, *p* = .001) and MDesat (r = –0.425, *p* = .011). Pegboard was significantly positively correlated with MinOxy(r = 0.460, *p* = .006) and MDesat (r = 0.440, *p* = .008), while being positively correlated at the trend level with TST (r = 0.297, *p* = .059). Pegboard was also significantly negatively correlated with % ≤88 (r = –0.423, p = .011) and pAHI (r = –0.499, *p* = .003).

Table 4 also presents the partial correlations between the WatchPAT sleep variables and depressive mood variables. In the full sample (n = 81), there was a significant negative correlation between TST and Reiss Depression-B (r = –0.552, *p* < .001), Reiss Depression-P (r = –0.440, *p* = .007), and the presence of a clinical depression diagnosis (r = –0.309, *p* = .046). Higher %Wake was also significantly positively associated with Reiss Depression-B (r = 0.313, *p* = .043). In follow-up analyses (n = 60) on the subset of 60 participants not using OSA treatment, there were no significant associations between depressed mood variables and WatchPAT variables.

## Discussion

The current study provided a preliminary investigation of the connection between sleep disruptions and OSA using an objective home sleep test and biomarkers of early AD pathology (PET Aβ and tau) and AD symptomatology (cognitive impairments and depressed mood symptoms). Findings highlight the high prevalence of OSA in adults with DS population. In our sample, 88 per cent (71 of 81) of the adults with DS were considered to have OSA—this includes the 60 adults with DS who screened positive on the WatchPAT home sleep test and 11 adults with DS with a prior medical diagnosis of OSA but who were successfully being managed with CPAP on the night of the home sleep test. Of the adults with DS who screened positive for OSA on the WatchPAT home sleep test, 44 per cent had moderate to severe OSA. These findings align with the prevalence of OSA reported in prior studies [4, 5]. Our findings also suggest that OSA often goes undetected in adults with DS. Indeed, 73 per cent of the adults with DS who did not have a prior medical diagnosis of OSA screened positive for OSA on the WatchPAT home sleep test. The severity of OSA on the WatchPAT did not differ between participants with and without a prior medical diagnosis of OSA, indicating that even moderate and severe cases of OSA often go undetected. Our findings also highlight that treating OSA may be difficult in adults with DS, as 50 per cent (11 of 22) of the adults with DS using OSA treatments during the home sleep study exhibited clinically significant sleep-disordered breathing problems with reports that CPAP devices were often removed during the night—a pattern that was also evident in the WatchPAT recordings.

There were important associations between sociodemographic variables and WatchPAT parameters. BMI was negatively associated with minimum oxygen level and positively associated with the number of apnea/hypopneas (i.e. pAHI level) during sleep. These findings remained when excluding the 21 participants who received OSA treatment during the WatchPAT home sleep test. In addition, when examining this smaller subset of 60 participants, there was a positive association between age and %Wake, indicating that sleep disruptions increase across adulthood. These findings align with broader literature in the general population [47, 48] and thus BMI Adults with DS with ApoE ε4 also had higher minimum oxygen levels, an unexpected finding given that ApoE ε4 is typically considered a risk factor. Future research should explore these associations in larger and more diverse samples.

Disrupted sleep and sleep-disordered breathing problems were related to AD pathology and symptomatology after adjusting for age, sex, and premorbid ID level in our sample of adults with DS. Specifically, a higher percentage of nighttime awakenings were associated with higher PET Aβ and tau burden, and there was a trend-level association between longer total sleep time and lower PET Aβ burden. These patterns remained true when examining the subset of participants not using OSA treatment during the home sleep study. In contrast, we did not find associations between sleep-disordered breathing variables and PET Aβ and tau, suggesting that disruptions in sleep may be more relevant to Aβ and tau production, deposition, and/or clearance than hypoxic events, which is in line with prior work in DS [49]. For example, longitudinal studies outside of DS have suggested that sleep fragmentation accelerates Aβ accumulation and is related to higher CSF T-tau/Aβ_42_ ratios [50]. However, it is important to note that our study was cross-sectional and cannot establish the causal direction of associations. While disrupted sleep may alter Aβ and tau production, deposition, or clearance mechanisms, it is also possible that elevated Aβ and tau burden disrupt sleep through mechanisms such as altering orexin levels and other sleep-related neurochemicals [51, 52]. Longitudinal research is needed to clarify the bidirectional relationship between sleep disruptions and AD pathology in individuals with DS.

Adults with DS with shorter total sleep time, higher percentages of time spent awake during the night, and more disrupted breathing (e.g. lower mean oxygen desaturation) exhibited poorer performance on executive functioning and motor planning and control tasks. Additionally, adults with DS who had an AD clinical status of MCI had greater OSA severity compared to those who were cognitively stable. Interestingly, WatchPAT sleep metrics were not related to memory impairments on the mCRT, which is often an early AD symptom. Thus, it could be that the effects of sleep disruptions and OSA are particularly harmful to executive functioning and motor planning and control. These findings suggest that sleep disruptions may contribute to declines in cognitive functioning and risk of MCI in the DS population. Shorter total sleep time and more time spent awake during the night were also associated with more depressive symptoms. The presence of a depression diagnosis by a health provider was also associated with shorter total sleep duration. Interestingly, depressive symptoms were not associated with indices of sleep-disordered breathing, suggesting that depressed mood symptoms in this population may be more closely linked to disruptions in sleep stages rather than to hypoxic events. All adults with DS diagnosed with depression were also diagnosed with OSA, underscoring the strong comorbidity between these two conditions.

### Strengths and limitations

This study has several notable strengths. It examined associations between sleep disruptions, OSA, and biomarkers of early AD pathology and symptoms using objective sleep measures. Moreover, the inclusion of neuroimaging biomarkers, direct cognitive measures, and informant-based assessments were used to assess AD pathology and symptomatology. However, there were also study limitations. Although the WatchPAT device has been validated against polysomnography in other populations, it has not been directly validated for use in adults with DS. Given the evidence of endothelial dysfunction in DS, it is possible that the device may be less sensitive in detecting certain sleep-disordered breathing events in this population [53]. Future studies should seek to validate portable home sleep tests specifically in adults with DS to improve the accuracy of at-home sleep assessments. The small-size and cross-sectional design of the study also limits the ability to draw causal inferences about the relationship between sleep disruptions and AD pathology. The alpha level for determining statistical significance was also not adjusted for multiple comparisons and thus findings are preliminary and should be interpreted with caution until replicated in larger samples.

Longitudinal studies are needed to clarify whether sleep disturbances contribute to the progression of AD or whether neurodegenerative changes associated with AD lead to sleep disruptions. Additionally, our sample was homogeneous in terms of race and ethnicity, and only three participants had transitioned to MCI. Larger, more diverse samples are needed to enhance the generalizability of our findings and to better understand the role of sleep problems in AD progression among adults with DS who have progressed to dementia. Finally, we included adults with DS (n = 22) who received OSA treatment during the night of the WatchPAT home sleep study as adults with DS often face barriers to adhering to CPAP treatment and thus may experience sleep problems despite having been prescribed these devices. However, follow-up analyses were conducted after excluding these adults with DS to ensure that connections between sleep and AD were not obscured.

In conclusion, our findings provide preliminary evidence that sleep disruptions and OSA may be underdiagnosed and undertreated in adults with DS. These findings suggest that incorporating routine screening for sleep problems into healthcare for adults with DS warrants further investigation as a potential strategy to mitigate risk. Larger longitudinal studies are needed to inform updated guidelines and recommendations for sleep assessment and management. Findings also add to the growing evidence of important connections between disrupted sleep and sleep-disordered breathing problems and the timing of AD pathology and symptomatology in adults with DS. However, findings from the current study need to be replicated in larger and longitudinal studies. Addressing sleep problems could represent an important intervention target for delaying the onset and progression of AD in the DS population.

## Data Availability

The data underlying this article are available at: https://pitt.co1.qualtrics.com/jfe/form/SV_cu0pNCZZlrdSxUN, the Alzheimer Biomarker Consortium-Down syndrome publicly available database. The data can be accessed using unique identifiers other than a DOI.

## Notes

For the Alzheimer’s Biomarkers Consortium–Down Syndrome (ABC-DS) Investigators.
